# An Infant with Xpert^®^ Confirmed TB Meningitis in Central Viet Nam

**DOI:** 10.3390/jcm7110397

**Published:** 2018-10-29

**Authors:** Phuong T. K. Nguyen, Trang T. B. Thai, Julie Huynh, Ben J. Marais

**Affiliations:** 1 Respiratory Department, Da Nang Hospital for Women and Children, 402 Le Van Hien Street, Ngu Hanh Son District, Da Nang 550000, Viet Nam; 2 Discipline of Paediatrics and Adolescent Medicine, The Children’s Hospital at Westmead, The University of Sydney, Westmead 2145, Australia; Julie.Huynh@health.nsw.gov.au (J.H.); ben.marais@health.nsw.gov.au (B.J.M.); 3 Intensive care unit, Da Nang Hospital for Women and Children, 402 Le Van Hien Street, Ngu Hanh Son District, Da Nang 550000, Viet Nam; trangthai_126@yahoo.com; 4 Department of Infectious diseases and Microbiology, The Children’s Hospital at Westmead, Westmead 2145, Australia; 5 Marie Bashir Institute for Infectious Diseases and Biosecurity, The University of Sydney, Westmead 2145, Australia

**Keywords:** tuberculosis, tuberculous meningitis, miliary, disseminated tuberculosis

## Abstract

A 5-month-old boy presented with a focal seizure. Disseminated (miliary) tuberculosis (TB) was diagnosed on chest radiograph and TB meningitis was confirmed using Xpert MTB/RIF^®^. The case represents the first instance of cerebrospinal fluid Xpert MTB/RIF^®^ testing in children in central Viet Nam. Family screening diagnosed the father with sputum smear-positive TB. The mother and a 2-year-old sibling had no symptoms or signs of TB disease and started preventive therapy. Early TB meningitis diagnosis is the single most important factor influencing clinical outcome, but is difficult due to the non-specific signs and symptoms at disease onset. Late diagnosis is associated with high mortality and severe neurologic handicap, which emphasizes the value of TB preventive therapy in vulnerable young children in close contact with an infectious TB case (recent is generally defined as within the last 12 months).

## 1. Introduction

A 5-month-old boy was transferred from a district hospital to a provincial referral hospital in central Viet Nam. He presented with a prolonged seizure that had a left-sided focal onset with secondary generalization, was given oxygen and diazepam, and was then transferred to the Da Nang Hospital for Women and Children. On admission the seizure had stopped, having lasted more than 30 min, and he was fully conscious. The mother noted that he had developed a low-grade fever and vomited thrice on the day prior to admission, following a period of poor feeding. He weighed 7.0 kg, and vital signs were normal. There were no signs of respiratory distress, with symmetrical breath sounds and no rales. The initial examination noted no focal neurological signs or bulging fontanel, but given his prolonged complicated seizure he was admitted to the intensive care unit (ICU) for close observation.

**Figure 1:**
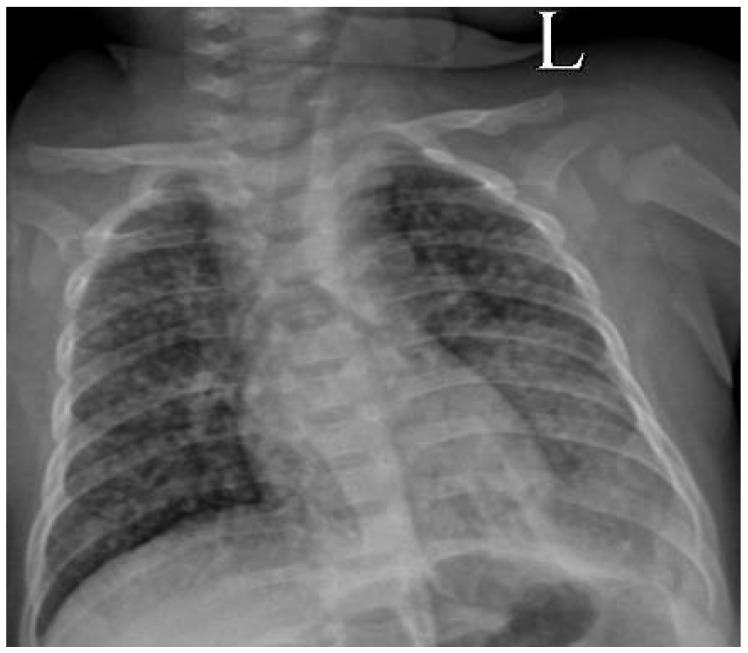
Chest radiograph on admission demonstrating bilateral nodular infiltrates (together with left lower lobe consolidation), suggestive of disseminated (miliary) tuberculosis.

Laboratory investigations were unremarkable for bacterial infection: the C-reactive protein (CRP) value was <6.0 mg/L and the procalcitonin (PCT) value was 0.3 ng/mL. His full blood count showed raised neutrophils (white blood cells 18.1 × 10^9^/L; neutrophils 13.3 × 10^9^/L), but this is not uncommon after a prolonged seizure. The chest radiograph demonstrated bilateral nodular interstitial infiltration, highly suspicious of disseminated (miliary) tuberculosis (TB), with opacification of the left lower lobe (Figure 1). A cranial ultrasound showed dilated lateral and third ventricles, consistent with hydrocephalus. The cerebrospinal fluid (CSF) was clear, with pleocytosis (207 cells/mm^3^, 75.0% neutrophils, 25.0% lymphocytes), increased protein (8.3 g/L), reduced glucose (0.7 mmol/L), and a positive Pandy test. Blood and CSF cultures were negative, but a Gene Xpert^®^ test performed on CSF was positive for *Mycobacterium tuberculosis* complex, without any *rpoB* mutations suggestive of rifampicin resistance. Gastric aspirate analysis for tuberculosis (TB) (including Gene Xpert^®^ and smear for acid-fast bacilli) was negative; no mycobacterial culture facilities were available. A rapid human immunodeficiency virus (HIV) test (HIV Combi PT, Roche, Mannheim, Germany) was negative. A computed tomography (CT) scan of the brain confirmed hydrocephalus together with basal meningeal enhancement. No visible infarcts were noted.

On day 4 of admission the patient was commenced on TB treatment for disseminated (miliary) TB and TB meningitis, including oral isoniazid, rifampicin, pyrazinamide and ethambutol, together with intramuscular streptomycin and intravenous (IV) dexamethasone (0.6 mg/kg/day). After 2 days of TB treatment his consciousness deteriorated (Glasgow Coma Scale (GCS) 8), requiring intubation and ventilation. By day 10 he showed no clinical improvement and a repeat chest radiograph showed progression of left lower and upper lobe consolidation. Blood and endotracheal tube aspirate cultures were negative, with normal inflammatory markers. He was commenced on IV ceftazidime and gentamycin, which was changed to IV meropenem and amikacin after 2 days when he showed no improvement. Antibiotics were continued for 14 days and he received IV mannitol, with ongoing sedation and ventilation, to control his raised intracranial pressure.

**Figure 2:**
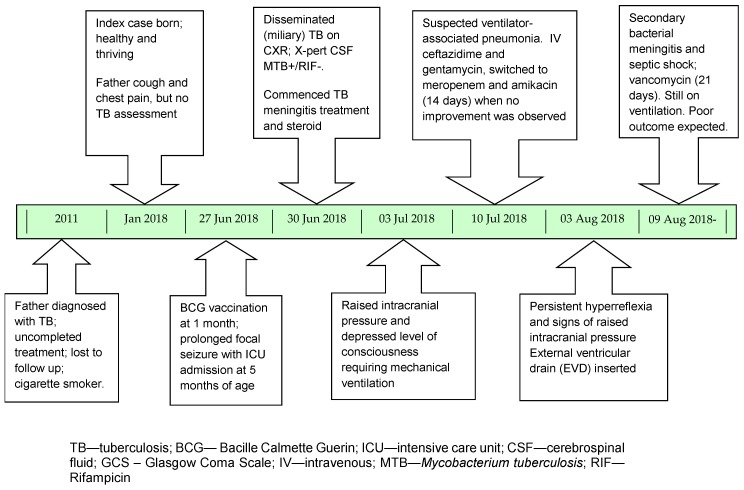
Timeline of relevant events and clinical progress.

After 1 month of TB treatment, his consciousness was still suppressed (GCS 10) with persistent hyperreflexia and clonic episodes. Following extra ventricular drain (EVD) insertion his fever settled and he regained full consciousness. Unfortunately, 6 days later he developed secondary bacterial meningitis with gram-positive cocci on CSF microscopy, and a positive Pandy test. Vancomycin and meropenem were used for 21 days. After nearly 2 months in hospital the patient remains mechanically ventilated with a poor prognosis (new infarcts and persistent hydrocephalus on CT scan). Characteristic head CT features of TB meningitis have been previously reviewed [1]. Figure 2 provides an overview of his clinical progress.

## 2. Additional Medical History

The infant was breastfed and thrived until the onset of poor feeding a few days before presentation. He received the Bacillus Calmette Guerin (BCG) vaccine at 1 month of age and had a BCG scar on his left upper arm. His father had been diagnosed with pulmonary TB 7 years earlier, but was “lost to follow up” before treatment completion. In recent months, he developed a worsening cough and left-sided chest pain. After the index case was diagnosed with TB, a chest radiograph revealed a large cavity in the father’s left upper lobe and he was found to be 3+ sputum smear-positive. He started on TB treatment at local TB hospital. The mother and the 2-year-old sibling were commenced on isoniazid preventive therapy in the absence of symptoms and normal chest radiographs.

## 3. Discussion

TB meningitis is the most devastating form of TB with high morbidity and mortality [2]. Early diagnosis and rapid initiation of appropriate TB meningitis treatment is the single most important determinant of disease outcome [2], but can be challenging to achieve [3]. However, hydrocephalus with CSF pleocytosis (especially if the cell count is <500 cells/mm^3^) are classic signs of TB meningitis [4]. CSF neutrophil predominance is not uncommon in the early phases of TB meningitis and concurrent CSF chemistry findings were highly suggestive [4,5].

The TB meningitis diagnosis was essentially confirmed when miliary infiltrates were observed on the CXR, but treatment was only initiated after the positive Xpert MTB/RIF^®^ test provided bacteriological confirmation. Our case represents the first instance of CSF Xpert MTB/RIF^®^ testing in children in central Viet Nam. The World Health Organization (WHO) endorsed the use Xpert MTB/RIF^®^ for TB meningitis detection and recently Xpert Ultra^®^ was shown to be even more sensitive [6]. In our case, the ability to exclude rifampin resistance was valuable, given that the father had recurrent TB. According to a recent survey, 23.3% of recurrent TB cases are multi-drug resistant (to isoniazid and rifampicin) in Viet Nam [7].

Taking a meticulous TB exposure history is essential at birth, as that could impact the timing of administration of BCG vaccination, acknowledging the stigma associated with TB in certain communities [8]. The father was the likely source case and preventive therapy could have protected the infant if his diagnosis had been known and contact tracing performed as per national TB guidelines. Prevention is of utmost importance, since only 12.0% of TB meningitis cases are diagnosed with stage 1 disease when a good outcome is most likely [9]. Our case was likely infected before BCG-induced immune responses offered any protection.

Although the infant was started on four TB drugs and steroids, the use of streptomycin can be questioned. Streptomycin does not penetrate the CSF and is associated with considerable ototoxicity; its use in children is actively discouraged in WHO child TB guidelines. Delayed treatment initiation and sub-optimal management of his raised intracranial pressure also adversely affected his outcome. Children with communicating hydrocephalus can often be medically managed (usually with acetazolamide and frusemide), but surgical CSF drainage is required if this fails or with non-communicating hydrocephalus. Optimal hydrocephalus management is a major dilemma in settings where imaging and surgical options are limited [10].

The patient in this case had advanced (stage 2) TB meningitis at presentation, with rapid progression to stage 3 disease. The reported mortality from TB meningitis in HIV-uninfected children is ~20.0% and the risk of neurological sequelae in those that survive is >50.0%. This risk is highest (70.0%) in stage 3 disease [11]. Corticosteroids reduce the risk of death by 25.0%, but offer little benefit to reduce neurological disability in survivors [12]. Stroke, mediated by vasculitis, may occur despite adequate TB treatment and is a major contributor to long-term morbidity [13]. Our understanding of the host inflammatory response remains limited and effective therapy to modulate this process has yet to be established. Xpert MTB/Rif^®^ availability increases diagnostic access, but there remains an urgent need to optimize prevention strategies and improve the clinical management of TB meningitis in children.

In conclusion, late diagnosis of TB meningitis is associated with high mortality and severe neurologic handicap. It is important to emphasize the value of TB preventive therapy in vulnerable young children with close TB contact.
